# Localization, Gene Expression, and Functions of Glutamine Synthetase Isozymes in Wheat Grain (*Triticum aestivum L*.)

**DOI:** 10.3389/fpls.2021.580405

**Published:** 2021-02-09

**Authors:** Yihao Wei, Shuping Xiong, Zhiyong Zhang, Xiaodan Meng, Lulu Wang, Xiaojiao Zhang, Meiqin Yu, Haidong Yu, Xiaochun Wang, Xinming Ma

**Affiliations:** ^1^Collaborative Innovation Center of Henan Grain Crops, College of Agronomy, Henan Agricultural University, Zhengzhou, China; ^2^Department of Biochemistry and Molecular Biology, College of Life Science, Henan Agricultural University, Zhengzhou, China

**Keywords:** glutamine synthetase, localization, nitrogen, glutamate dehydrogenase, wheat, grain

## Abstract

Glutamine synthetase (GS) plays a major role in plant nitrogen metabolism, but the roles of individual GS isoforms in grains are unknown. Here, the localization and expression of individual TaGS isozymes in wheat grain were probed with TaGS isoenzyme-specific antibodies, and the nitrogen metabolism of grain during the grain filling stage were investigated. Immunofluorescence revealed that TaGS1;1, TaGS1;3, and TaGS2 were expressed in different regions of the embryo. In grain transporting tissues, TaGS1;2 was localized in vascular bundle; TaGS1;2 and TaGS1;1 were in chalaza and placentochalaza; TaGS1;1 and TaGS1;3 were in endosperm transfer cells; and TaGS1;3 and TaGS2 were in aleurone layer. GS exhibited maximum activity and expression at 8 days after flowering (DAF) with peak glutamine content in grains; from then, ${\text{NH}}_{4}^{+}$ increased largely from ${\text{NO}}_{3}^{-}$ reduction, glutamate dehydrogenase (GDH) aminating activity increased continuously, and the activities of GS and glutamate synthase (GOGAT) decreased, while only TaGS1;3 kept a stable expression in different TaGS isozymes. Hence, GS-GOGAT cycle and GDH play different roles in ${\text{NH}}_{4}^{+}$ assimilation of grain in different stages of grain development; TaGS1;3, located in aleurone layer and endosperm transfer cells, plays a key role in Gln into endosperm for gluten synthesis. At 30 DAF, grain amino acids are mainly transported from maternal phloem.

## Introduction

Nitrogen (N) is a vital macronutrient for crops, which is essential for crop growth and a primary driver of grain yield (Hirel et al., 2007; Xu et al., 2012). To increase grain yield, N fertilizers are applied excessively, leading to severe environmental pollution (Robertson and Vitousek, 2009; Kant et al., 2010). Hence, there is an urgent demand to optimize the usage of N fertilizers to make agriculture environmentally friendly. One of the optimization methods is to improve the crop N use efficiency (NUE), which is defined as the grain yield per unit of applied N fertilizer (McAllister et al., 2012; Xu et al., 2012; Thomsen et al., 2014).

To improve crop NUE, glutamine synthetase (GS; EC 6.3.1.2) has been identified as one of the main objects of crop research, owing to its essential role in the assimilation of ammonium (${\text{NH}}_{4}^{+}$) (Martin et al., 2006; Hirel et al., 2007; Bernard et al., 2008; Nigro et al., 2016; Zhang et al., 2017; Hu et al., 2018; Gao et al., 2019). GS catalyzes the adenosine triphosphate (ATP)-dependent fixation of ammonium (${\text{NH}}_{4}^{+}$) into the glutamate (Glu) to form glutamine (Gln). The enzyme glutamate synthase (GOGAT) catalyzes the conversion of Gln and 2-oxoglutarate to two molecules of Glu, one molecule serves as a substrate for GS and the other participates in the synthesis process of various N-containing compounds (Bernard and Habash, 2009). In plants, GSs are classified into two groups based on their subcellular locations: the cytosolic glutamine synthetase isoform (GS1) and the plastidic glutamine synthetase isoform (GS2). Generally, GS1 is encoded by 3–5 nuclear genes, while GS2 is encoded by one nuclear gene (Swarbreck et al., 2010).

Studies on quantitative trait locus (QTL) analysis, knockout, and overexpression of GS have demonstrated that it is closely related to the grain yield of crops. In rice, the QTL for single spikelet yield colocalizes with *OsGS1* (Obara et al., 2001). The *OsGS1;1* knockout mutants showed a significant decrease in both number and weight of rice spikelet (Tabuchi et al., 2005). In maize, *ZmGln1;3* colocalizes with QTL region for grain yield and thousand grain weight (Gallais and Hirel, 2004). The knockout mutant of *ZmGln1;3* and *ZmGln1;4* results in reduced kernel number and kernel size, respectively (Martin et al., 2006). In wheat, transgenic expression of *TaGS2-2Ab* increased ear spike number, grain number per spike, and 1,000-grain weight (Hu et al., 2018). In barley, overexpression of *HvGS1;1* improved the grain yield and NUE (Gao et al., 2019). In Arabidopsis, the knockout mutant of *AtGln1;2* results in decreased seed yield due to a reduction in the number of siliques, less seeds per silique, and lower dry weight per seed, indicating that *AtGln1;2* plays an important role in seed yield structure (Guan et al., 2015).

Grain yield requires the joint participation of different tissues, and GS plays different functions in different tissues. In roots, GS1 participates in primary ${\text{NH}}_{4}^{+}$ assimilation and Gln transportation (Ishiyama et al., 2004a,b; Martin et al., 2006; Konishi et al., 2017), and GS2 may participate in assimilating ${\text{NH}}_{4}^{+}$ derived from ${\text{NO}}_{3}^{-}$ reduction (Goodall et al., 2013; Wei et al., 2018). In leaves, GS1 is important to re-assimilate ${\text{NH}}_{4}^{+}$ generation during protein turnover and to transport N to sink in the form of Gln (Martin et al., 2006; Bernard et al., 2008; Moison et al., 2018), whereas the dominating role of GS2 is to assimilate ${\text{NH}}_{4}^{+}$ derived from photorespiration and ${\text{NO}}_{3}^{-}$ reduction (Wallsgrove et al., 1987; Thomsen et al., 2014; Wang et al., 2015). In stems, GS1 plays a role in transportation N in the form of Gln; the functions of GS2 are similar to it in leaves (Bernard et al., 2008).

For crops, grains are the most important storage tissue. The process of assimilates entering a grain is different from that in other tissues. Grain filling relies on the unhampered importation of assimilates from maternal tissues into filial tissues (Wang et al., 1995). However, there is no direct vascular bundle (VB) linked between maternal and filial tissues (Bagnall et al., 2000). In maize, assimilates derived from VB are unloaded at the vascular bundle terminal (VBT) to the placentochalaza region and then absorbed into endosperm by endosperm transfer cells (ETC) (Purcino et al., 2000; Thompson et al., 2001; Zheng, 2012). In wheat, the assimilates from the maternal VB are passed through the chalazal region into the endosperm cavity (EC) and then transported by ETC into the endosperm (Wang et al., 1994; Patrick and Offler, 2001; Thompson et al., 2001). In developing wheat grains, ${\text{NH}}_{4}^{+}$ is mainly assimilated through combined action of glutamine synthetase, glutamate synthase (GOGAT), and glutamate dehydrogenase (GDH) (Garg et al., 1984). GS and GOGAT localized in the testa-pericarp play a dominant role in ${\text{NH}}_{4}^{+}$ assimilation during the early stages of grain development, and GDH localized in the endosperm plays a more active role during the later stages (Garg et al., 1984, 1985).

In cereals, GS isozymes have high homology according to phylogenetic tree analysis of individual GS isoforms of cereals (Thomsen et al., 2014). Thomsen et al. (2014) clustered them into four categories: GS1;1, GS1;2, GS1;3, and GS2. In wheat, TaGS isozymes are classified into four subfamilies: TaGS1, TaGSr, TaGSe, and TaGS2 (Bernard et al., 2008). Based on the clustering of TaGS isoforms, we re-named TaGS1, TaGSr, and TaGSe genes as TaGS1;1, TaGS1;2, and, TaGS1;3, respectively. Although GS activity is detected only in the testa-pericarp of wheat grains (Garg et al., 1985), no previous research has been reported on the distribution of individual TaGS isoforms in the grain or the role of individual TaGS in nitrogen assimilation or transportation in grains during the filling stage, which may be important to improve the grain yield. To solve this problem, we designed specific antibodies to TaGS1;1, TaGS1;2, TaGS1;3, and TaGS2. By investigating cellular localization and expression profiles of TaGS isozymes in grains with specific antibodies as the probe and studying the characteristics of N metabolism during the grain filling stage, we clarify the role of individual TaGS isoforms during wheat grain development.

## Materials and Methods

### Wheat Growth Conditions and Experimental Design

The field experiment was conducted during the 2017–2018 growing season at the experimental station of Hua county, Henan, China (114°37′37.8″E, 35°31′06.6″N). The Hua county site is in the center of China and has a warm temperate continental monsoon climate. Meteorological data, including air temperature and rainfall, for growth seasons were acquired from the meteorological station of Hua county (Supplementary Table 1). The previous crop was maize. The experiment was conducted in field plot, with a size of 6 m × 2.8 m, repeated three times. Seeds were sown in rows at 20-cm spacing, with 14 rows per plot at a density of 270 plants m^−2^. The clay soil contained 12.13 g organic matter kg^−1^, 1.32 g total N kg^−1^, 91.32 mg water-hydrolyzable N kg^−1^, 74.25 mg rapidly available phosphate kg^−1^, and 236 mg rapidly available potassium kg^−1^. Urea (46.4% N) was used as a nitrogenous fertilizer to perform the experiment, and the total N supply was 225 kg ha^−1^. Sixty percent of the total N was directly mixed into the soil and was combined with calcium superphosphate (120 kg ha^−1^) and potassium chloride (120 kg ha^−1^) before plowing, and 40% was dissolved in water and applied to the soil at the elongation stage. Irrigation was carried out in the overwintering stage and stem elongation stage. The wheat cultivar Yumai 49 (YM49) is one of the varieties popularized in Henan province of China, which has the characteristics of high and stable yield and high nitrogen use efficiency. Seeds of YM49 were purchased from Henan Pingan Seed Co., Ltd (Jiaozuo, China). Seeds were sown on 30 September 2017 and plants were harvested on 16 June 2018.

The flowering stage was defined as when > 50% of the spikes within a plot showed protruding anthers. To track days after flowering (DAF), the main stems were labeled at anthesis. At 8, 16, 24, and 30 DAF, grain material in the middle of spike from 20 main stems was mixed as a sample and frozen in liquid N for 3 h and stored at −80°C for the measurement of physiological indicators (N metabolite and enzyme activity). Two samples were collected from each of the three plots for a total of six replicates. At 16 DAF, grains in the middle of the spike on the main stem were sampled and immediately immersed in fixative for the immunolocalization studies.

### Cloning of Wheat GS cDNAs

The sequences of wheat GS cDNAs were isolated by RT-PCR. The wheat variety YM49 was used to isolate *TaGS1;1, TaGS1;2, TaGS1;3*, and *TaGS2* sequences. Total RNA was extracted from the leaves, stems, and seeds using TRIzol Reagent (Thermo Scientific, MA, United States) following the manufacturer's instructions. Reverse transcription and PCR were carried out using the HiScriptII1st Strand cDNA Synthesis Kit (Vazyme Biotech Co., Ltd., Nanjing, China) and Phanta EVO Super-Fidelity DNA Polymerase (Vazyme). Primers were designed according to the wheat GS cDNAs in the NCBI databases (Supplementary Table 2). The amplified PCR products were cloned into pMD19-T Simple Vector (TaKaRa Biotechnology Co., Ltd., Dalian, China) and fully sequenced.

### Design and Production of Antibodies Against Individual Wheat GS Isozymes

**Figure 1:**
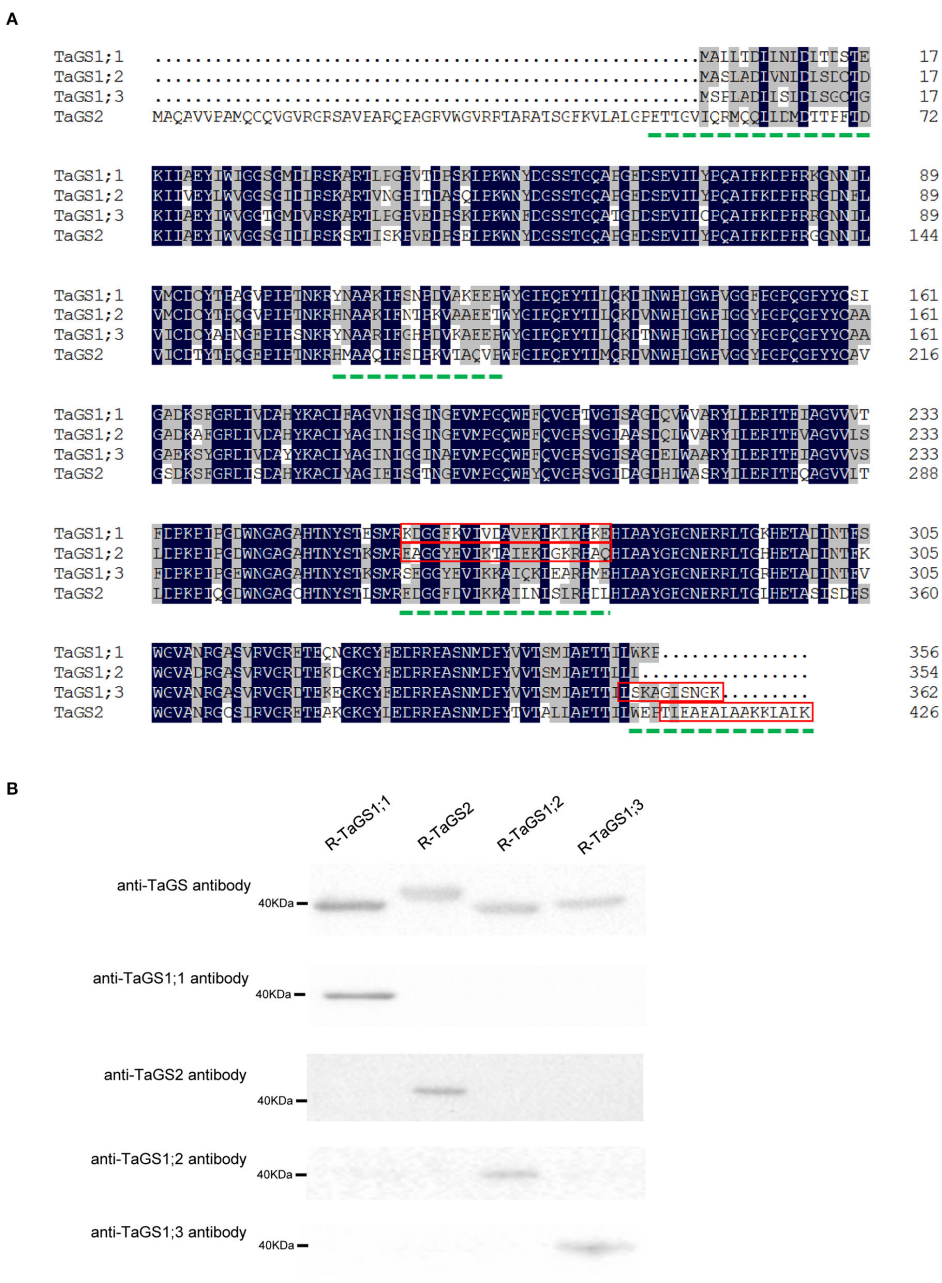
Polypeptide sequences selected as antigen and specificity analysis of the prepared antibodies. **(A)** Multiple alignment of wheat glutamine synthetase amino acid sequences. The dotted green line indicates low homology amino acid sequence of TaGS1;1, TaGS2, TaGS1;2, and TaGS1;3. The polypeptide sequences selected as antigenic based on antigenic, hydrophilic, and surface accessibility are highlighted by a red box. **(B)** The specificity of anti-GS antibodies to the individual recombinant TaGS subunits (R-TaGS). The dilution ratios of the anti-TaGS, anti-TaGS1;1, anti-TaGS2, anti-TaGS1;2, and anti-TaGS1;3 antibody are 1:5,000, 1:30,000, 1:10,000, 1:30,000, and 1:10,000, respectively. About 2.5 μg of soluble proteins extracted from the *E. coli* were loaded onto each lane.

MegAlign (DNASTAR Inc., Madison, WI, USA) was used to compare the amino acid sequences of TaGS1;1, TaGS1;2, TaGS1;3, and TaGS2 (Figure 1A). The hydrophilicity, surface accessibility, and antigenicity of polypeptide sequences with low homology were analyzed with Protean (DNASTAR) (Table 1). The polypeptide sequences with strong antigenic, hydrophilic, and surface accessibility were selected as antigenic of individual wheat GS isozymes, i.e., TaGS1;1: KDGGFKVIVDAVEKLKLKHKE; TaGS1;2: EAGGYEVIKTAIEKLGKRHAQ; TaGS1;3: LSKAGLSNGK; and TaGS2: TLEAEALAAKKLALKV. The synthesis of these antigenic polypeptides and the subsequent immunization of the rabbit were completed by Zoonbio (Zoonbio Biotechnology Co., Ltd., Nanjing, China). The specificity of antibodies was detected by using individual recombinant TaGS proteins expressed in *Escherichia coli*.

**Table 1:** The antigenicity, surface accessibility, and hydrophilicity of polypeptides with low homology of TaGS1;1, TaGS1;2, TaGS1;3, and TaGS2.

**Name**	**Sequence**	**Length (AA)**	**Antigenicity**	**Surface accessibility**	**Hydrophilicity**
TaGS1.1-1	MALLTDLLNLDLTDSTE	17	0.13	0.87	−0.34
TaGS1.1-2	YNAAKIFSNPDVAKEEP	17	0.37	1.35	0.56
**TaGS1.1-3**	**KDGGFKVIVDAVEKLKLKHKE**	21	0.87	1.22	0.24
TaGS1.2-1	MASLADLVNLDLSDCTD	17	0.57	0.48	−0.44
TaGS1.2-2	HNAAKIFNTPKVAAEET	17	0.21	1.01	0.31
**TaGS1.2-3**	**EAGGYEVIKTAIEKLGKRHAQ**	21	0.43	1.1	0.52
TaGS1.3-1	MSPLADLLSLDLSGCTG	17	0.07	0.38	−0.7
TaGS1.3-2	SEGGYEVIKKAIQKLEARHME	21	0.61	1.14	0.64
**TaGS1.3-3**	**LSKAGLSNGK**	10	1.16	0.87	0.23
TaGS2-1	PETTGVIQRMQQLLD	15	0.01	0.98	0.35
**TaGS2-2**	**TLEAEALAAKKLALKV**	16	0.28	0.67	−0.44

***Note:** Protean (DANSTAR) software was used to predict antigenicity, surface accessibility, and hydrophilicity of polypeptides by Jameson–Wolf Method, Emini Method, and Kyte–Doolittle Method, respectively. The polypeptide sequences selected as antigenic are indicated in bold text*.

### Expression of Recombinant Wheat GS Protein in *E. coli*

We used the wheat GS cDNAs from wheat variety Yumai49 as the template and amplified the CDS (coding sequence) region for *TaGS1;1, TaGS1;2, TaGS1;3*, and *TaGS2* cDNA by PCR with the specific primers (Supplementary Table 3). The PCR products were cloned into the pET21a vector (Novagen) and fully sequenced. The recombinant vectors were constructed using ClonExpress One Step Cloning Kit (Vazyme). The CDSs of wheat GS were cloned into the *Nde* I and *Hind* III sites of pET21a vector (Novagen). The recombinant vectors and empty pET21a vector were transformed into Rosetta (DE3) pLysS cell. Protein production was induced by the addition of IPTG (isopropyl-b-D-thiogalactoside) to a final concentration of 1 mM and incubation in a shaker at 180 rpm. TaGS1;1, TaGS1;2, TaGS1;3, and TaGS2 were induced at 30°C for 5 h, 12°C for 17 h, 37°C for 5 h, and 25°C for 7 h, to obtain soluble protein. After induction, cells were harvested by centrifugation at 5,000*g* for 10 min at 4°C. The pellet was suspended in breaking buffer [10 mM Tris, 10 mM MgCl_2_, 0.05% Triton X-100, 100 μg ml^−1^ phenylmethanesulfonyl fluoride (PMSF), pH 7.5] and sonicated using an ultrasonic homogenizer JY92-2D (Ningbo Scientz Biotechnology Co. Ltd., Ningbo, China). The lysate was centrifuged at 12,000*g* for 15 min at 4°C and the supernatants were collected and used for identification of TaGS specific antibodies by western blot.

### Cellular Localization of TaGSs Using Immunofluorescence Analysis

At 16 DAF, grains were fixed in FAA fixative for at least 24 h. Grains were dehydrated in a graded alcohol series (75, 85, 90, 95, 100, and 100%) and in a mixture of 100% alcohol and 100% xylene (1:1, *v*/*v*), followed by two changes of pure xylene. Samples were incubated in three changes of paraffin at 65°C (1 h each) and embedded in paraffin. Sections (4 μm) were prepared using a microtome and dried on glass slides at 60°C.

Samples on slides were dewaxed and rehydrated by incubating sections in two changes of xylene for 15 min each. They were dehydrated in two changes of pure ethanol for 5 min, followed by dehydration in gradient ethanol of 85 and 75% ethanol, respectively, for 5 min each, and washed in distilled water. After, the slides were immersed in ethylenediaminetetraacetic acid (EDTA) antigen retrieval buffer (pH 8.0) (Wuhan Servicebio Technology Co., Ltd., Hubei, China) and maintained at sub-boiling temperature for 8 min, standing for 8 min, and followed by another sub-boiling temperature for 7 min and left to cool in the air. After three washes of 5 min each in phosphate-buffered saline (PBS) (pH 7.4), spontaneous fluorescence quenching reagent was added before incubation for 5 min. Then, slides were washed in running tap water.

Samples were blocked with 3% (*w*/*v*) bovine serum albumin in PBS (pH 7.4) (blocking solution) for 30 min and then incubated with the monospecific antibody diluted in blocking solution overnight at 4°C. Anti-TaGS1;1, anti-TaGS1;2, anti-TaGS1;3, and anti-TaGS2 antibodies were diluted at 1:200, 1:200, 1:500, and 1:50, respectively, in blocking solution. After three washes of 5 min each in PBS (pH 7.4), slides were incubated with goat anti-rabbit IgG labeled with FITC (Servicebio) in blocking solution at room temperature for 50 min in the dark. After three washes of 5 min each in PBS (pH 7.4), slides were incubated with DAPI (Servicebio) solution at room temperature for 10 min and kept in a dark place. After washing three times, we discarded some liquid and covered them with anti-fade mounting medium (Servicebio). Negative controls were conducted by substituting the TaGS monospecific antibodies with pre-immune rabbit serum. Microscopy detection and images were collected with fluorescent microscopy (Nikon Co., Ltd., Tokyo, Japan). DAPI glowed blue by UV excitation wavelength 330–380 nm and emission wavelength 420 nm; FITC glowed green by excitation wavelength 465–495 nm and emission wavelength 515–555 nm.

### RNA Isolation and Quantitative Real-Time PCR

Total RNA was extracted from plant tissue using HiPure HP Plant RNA Kit B (Guangzhou Magen Biotechnology Co. Ltd., Guangzhou, China). cDNA was synthesized using the RTIII Super Mix with dsDNase (Monad Biotech Co., Ltd, Shanghai, China). Quantitative real-time PCR (qPCR) was performed on a StepOne Real-Time PCR System (Life Technologies Corporation, Carlsbad, CA, United States) with SYBR Green qPCR Mix (Monad) for the assay. All primers (Sangon Biotech Co., Ltd., Shanghai, China) used are shown in Supplementary Table 4. The qPCR mix was composed of 10 μl SYBR Green qPCR Mix (Monad), 5 μl diluted cDNA 1:10 (*v*/*v*), 0.5 μl and 10 μM forward and reverse primers, respectively, and 4 μl of sterile nuclease-free water. Reactions proceeded according to the following program: 95°C for 10 min, followed by 40 cycles of 95°C for 15 s, 58°C for 15 s, and 72°C for 20 s. Fluorescence readings were taken during the elongation step (72°C). Melting curves were obtained from 60 to 95°C with a 0.5°C increase every 15 s. The *TaATPases* (Ta54227) and *TaTEF* (Ta53964) genes were used as reference genes (Paolacci et al., 2009). The geometric mean of Ct values of *TaATPases* and *TaTEF* served to normalize the expression ratio for each gene. Relative expression levels of genes were calculated *via* the 2^∧(−ΔΔCt)^ method.

### Extraction of Proteins From Wheat Grains and Western Blot Analysis

Approximately 0.3 g of fine homogeneous powder was mixed with 0.9 ml of GS extraction buffer (100 mM Tris, 1 mM EDTA, 1 mM MgCl_2_, 1 mM PMSF, and 10 mM β-mercaptoethanol; pH 7.6) by shaking at 4°C for 10 min. The extract was centrifuged at 12,000× *g* at 4°C for 30 min. The supernatant was then prepared for further experiments.

A total of 20 μg of soluble protein that was extracted from the grain was loaded onto each lane. Proteins were separated in 12.5% (*w*/*v*) polyacrylamide gel and electrophoretically transferred to a 0.45-μm pore size PVDF membranes (Merck Millipore Ltd., Darmstadt, Germany) in transfer buffer (25 mM Tris-base and 192 mM Gly, 10% methanol) at 200 mA for 50 min. The membranes were blocked with TBST [20 mM Tris-base, 150 mM NaCl, and 0.05% (*v*/*v*) Tween 20, pH 7.4] containing 5% skimmed milk at 4°C overnight. The membrane was incubated at 20°C for 1.5 h with the TaGS1;1, TaGS1;2, TaGS1;3, TaGS2, and HSP70 antibody, and the dilution ratio of the antibody was 1:5,000, 1:10,000, 1:30,000, 1:30,000 1:10,000, and 1:50,000, respectively. After three washes with TBST, the membrane was incubated at room temperature for 1 h with horseradish peroxidase-conjugated goat anti-rabbit IgG (ABclonal Biotechnology Co., Ltd., Hubei, China) at 1:25,000. After several washes with TBST, the membrane was incubated at room temperature for 5 min using Clarity Western ECL reagent (Bio-Rad) and the signals were detected by ChemiDoc^TM^ XRS^+^ Imaging System (Bio-Rad).

### Enzyme Activity and N Metabolite Analysis

The total GS activity was measured in accordance with the method described by Ma et al. (2005). One unit of GS activity was the enzyme catalyzing the formation of l μmol γ-glutamylhydroxamate/min at 37°C, and the whole GS activity was determined by the micromole sum of γ-glutamylhydroxamate catalyzed by the whole enzyme per gram fresh material in 1 min. NR activity was measured as described by Chen and Zhang (1980) except that the extraction buffer was the same to GS. The NR activity was expressed as micromole ${\text{NO}}_{2}^{-}$ generated per hour and gram fresh weight. GDH activity was measured as described by Turano et al. (1996) except that the extraction buffer was the same to GS. The GDH aminating activity was expressed as micromole NADH used per minute and gram fresh weight. The GOGAT activity with methyl viologen or NADH was assayed by determining Glu formation with high-performance liquid chromatography (HPLC), as described in Lancien et al. (2002). The Fd-GOGAT activity and NADH-GOGAT activity were expressed as micromole Glu generated per minute and gram fresh weight.

Free ammonium and nitrate content were measured according to Wei et al. (2018). Soluble sugar was measured using the anthrone colorimetric method (Tang, 1999). Glutenin content of grain was measured as described by Xu et al. (2003).

### Free Amino Acid Analysis Using HPLC

A fine homogeneous powder (0.1 g) was extracted with 1 ml H_2_O at 25°C for 12 h. The extracts were centrifuged and the supernatants were derivatized with triethylamine acetonitrile solution and phenyl isothiocyanate acetonitrile solution at 25°C for 12 h. HPLC was performed using a RIGOL L3000 Liquid Chromatograph (RIGOL Technology Co., Ltd., Suzhou, China). After derivatization, an amount equivalent to 10 μl of each sample was injected on a Kromasil C18 reversed phase column, 5 μm, 250 mm × 4.6 mm (Akzo Nobel N.V., Amsterdam, Netherlands), at 40°C, with detection at λ = 254 nm. Eluent A was 0.1 M sodium acetate prepared with 7% acetonitrile, adjusted to pH 6.5 with glacial acetic acid; eluent B was acetonitrile/water (80:20). The elution protocol was 0–2 min, 100% A; 2–15 min, linear gradient to 10% B; 15–25 min, linear gradient to 30% B; 25–33 min, linear gradient to 45% B; 33–38 min, 100% B; 38–45 min, 100% A. Results are means of six independent experiments.

### Phloem Exudate Collection

Phloem exudate collection from peduncle was done according to Simpson and Dalling (1981) with some modifications. At 8, 16, 24, and 30 DAF, the phloem exudates of peduncles on the main stems were collected. The spike was cut off and the peduncle recut under water before rapid immersion in the collection buffer. The exudates from peduncle to spike were collected. For each experiment, peduncle was placed in 1 ml solution of 10 mM HEPES and 10 mM EDTA (adjusted to pH 7.5 with NaOH) in a humid chamber in the dark. Exudates were collected during 4 h from 12:00 to 16:00. The method to obtain the saps is explained in Supplementary Figure 1. The amino acid content and component of phloem exudate were analyzed with HPLC.

### Statistics

All data represent the mean ± standard deviation (SD) of six biological replicates. The data sets were analyzed using Microsoft Excel (2016, Microsoft, Redmond, WA, USA). Data were statistically analyzed using SPSS version 13.0 (IBM, Chicago, IL, USA). One-way analysis of variance (ANOVA) with a Duncan *post-hoc* test was used to test statistical differences.

## Results

### Identification of Individual TaGS Isozyme-Specific Antibodies

To obtain specific antibodies of individual TaGS isozymes, the specific peptides of individual TaGS isozymes were used as antigens to immunize rabbits. The specificity of antibodies was detected by using individual recombinant TaGS proteins expressed in *E. coli*.

The individual recombinant TaGS protein loaded in the gel was adjusted to a uniform level, and the specificity of the antibodies against individual TaGS isozyme was tested. The results showed that anti-TaGS1;1, anti-TaGS1;2, anti-TaGS1;3, and anti-TaGS2 antibodies were monospecific to TaGS1;1, TaGS1;2, TaGS1;3, and TaGS2 polypeptides, respectively, with an antibody dilution ratio of 1:30,000, 1:30,000, 1:10,000, and 1:10,000, respectively (Figure 1B). The original images of western blot are shown in Supplementary Figure 2.

### Cellular Localization of TaGS Isozymes in the Grain of Wheat

To understand the roles of TaGS isozymes in grains, cellular localization was determined with immunofluorescence. As there is high sequence similarity among these isozymes, the immunofluorescence analysis was done with the monospecific antibodies.

**Figure 2:**
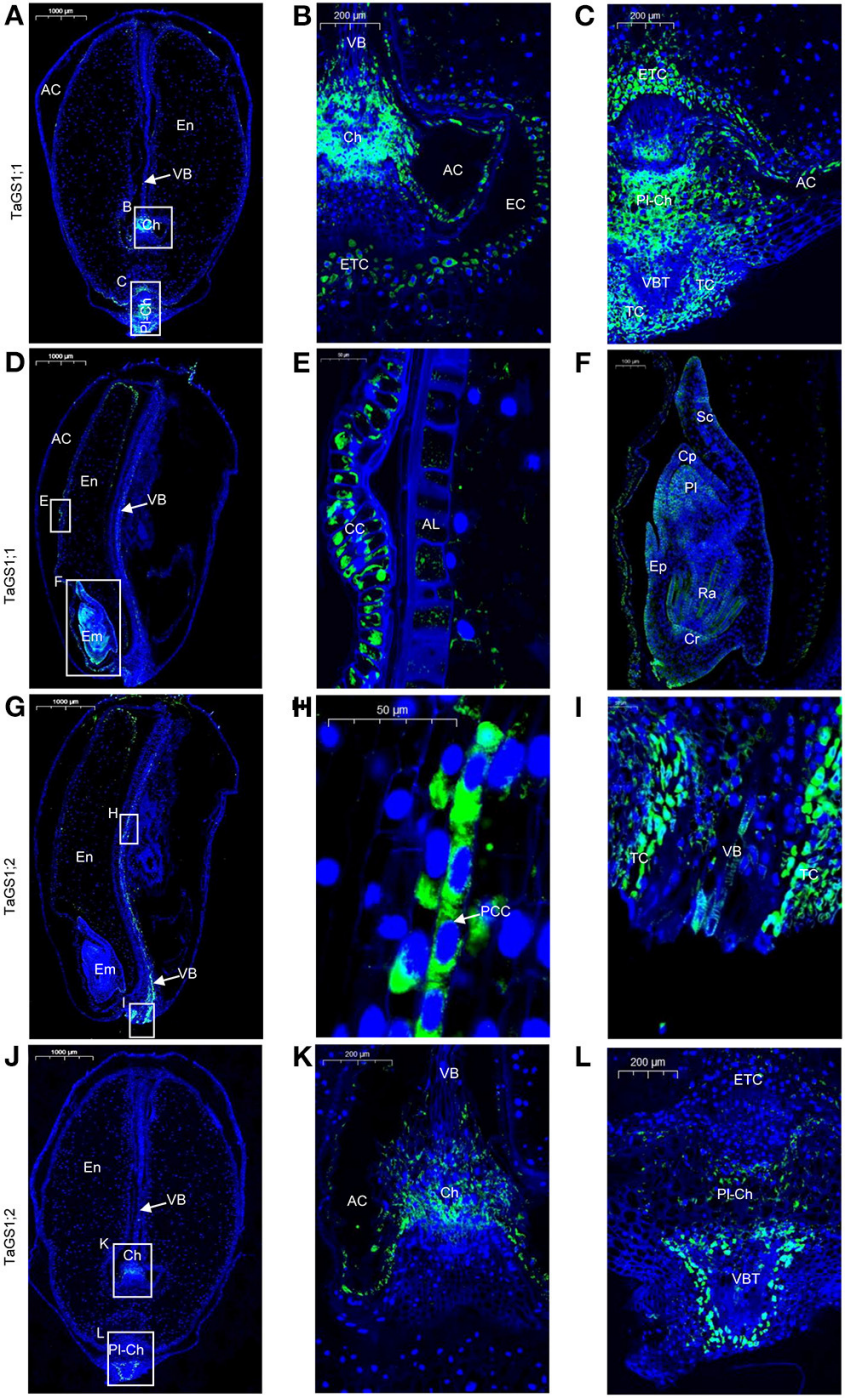
Cellular localization of TaGS1;1 and TaGS1;2 in grain sections at 16 days after flowering (DAF). Histological immunolocalization of TaGS1;1 in grain longitudinal section **(A–F)**. Magnification of the zone containing chalazal region **(B)**, placentochalaza region (**C**), aleurone layer **(E)**, and embryo **(F)**. Histological immunolocalization of TaGS1;2 in grain longitudinal section (**G–L**). Magnification of the zone containing vascular bundle **(H,I)**, chalazal region **(K)**, and placentochalaza region **(L)**. Em, embryo; En, endosperm; CC, cross cells; AL, aleurone layer; Cp, coleoptile; Cr, coleorhiza; Pl, plumule; Ra, radicle; Sc, scutellum; Ep, epiblast; TC, transfer cell; VB, vascular bundle; VBT, vascular bundle terminal; PCC, phloem companion cells; Ch, chalaza; ETC, endosperm transfer cells; Pl-Ch, placentochalaza; AC, apoplastic cavity; EC, endosperm cavity.

During wheat grain development, assimilates derived from the maternal VB are passed through the chalazal region into the EC and are then absorbed by ETC into the endosperm. In the wheat grain longitudinal section at 16 days after flowering (DAF), the VB, chalazal region, ETC, and endosperm were observed (Figures 2A,B). Moreover, a similar placentochalaza region between the vascular bundle terminal (VBT) and ETC (Figures 2A,C) was also observed in the wheat grain. This structure is generally found in the grain of maize, and assimilates derived from VB are unloaded at the VBT to the placentochalaza and are then absorbed into the endosperm by ETC (Thompson et al., 2001; Zheng, 2012).

In the wheat grain, green fluorescence signals corresponding to four different TaGS isozymes were detected. TaGS1;1 was distributed in the chalaza, endosperm transfer cells (Figure 2B), the placentochalaza, endosperm transfer cells and transfer cells around the VBT (Figure 2C), and cross cells (Figures 2D,E). It also has a wide expression in the embryo, mainly located in the coleoptile, plumule, radicle, coleorhiza, and epiblast (Figure 2F). However, TaGS1;2 was not detected in the embryo. It was mainly expressed in phloem companion cells of VB (Figures 2G,H), the transfer cell around the VB (Figures 2G,I), the chalaza (Figures 2J,K), the placentochalaza, and the cells next to the VBT (Figures 2J,L). The negative control section treated with pre-immune rabbit serum showed that no green fluorescence signal was detected in the grain sections (Supplementary Figure 3).

**Figure 3:**
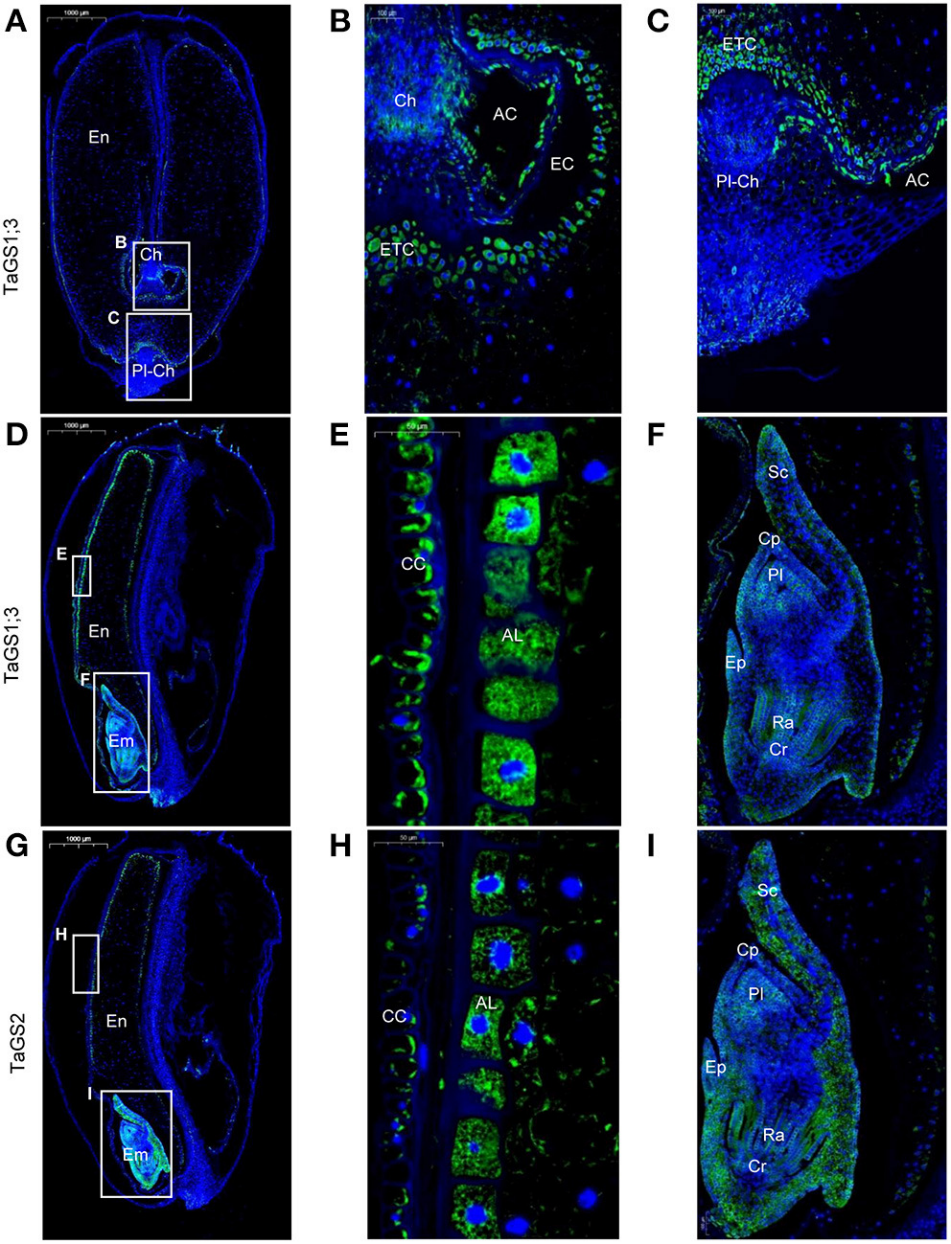
Cellular localization of TaGS1;3 and TaGS2 in grain sections at 16 DAF. Histological immunolocalization of TaGS1;3 in grain longitudinal section **(A–F)**. Magnification of the zone containing chalazal region **(B)**, placentochalaza region **(C)**, aleurone layer **(E)**, and embryo **(F)**. Histological immunolocalization of TaGS2 in grain longitudinal section (**G–I**). Magnification of the zone containing aleurone layer **(H)** and embryo **(I)**. Em, embryo; En, endosperm; CC, cross cells; AL, aleurone layer; Cp, coleoptile; Cr, coleorhiza; Pl, plumule; Ra, radicle; Sc, scutellum; Ep, epiblast; Ch, chalazal; ETC, endosperm transfer cells; Pl-Ch, placentochalaza; AC, apoplastic cavity; EC, endosperm cavity.

TaGS1;3 was mainly distributed in the endosperm transfer cells (Figures 3A–C) and aleurone layer cells (Figures 3D,E) and was also distributed in cross cells (Figure 3E). In addition, the localization of TaGS1;3 in the embryo is not only similar to that of TaGS1;1 but it was also distributed in the scutellum and scutellum epithelium cell (Figures 3D,F). TaGS2 was distributed in the cross cells and aleurone layer cells (Figures 3G,H). It was also widely distributed in embryos, but mainly in the edge of embryos such as scutellum (Figure 3I).

The cellular localization of protein is essential to judge its function. The distinct cellular localizations of individual TaGS in the grain suggest that they have different roles in N metabolism of grain.

### The Expression Profiles of TaGS Isozymes in the Grain During Grain Filling Stage

**Figure 4:**
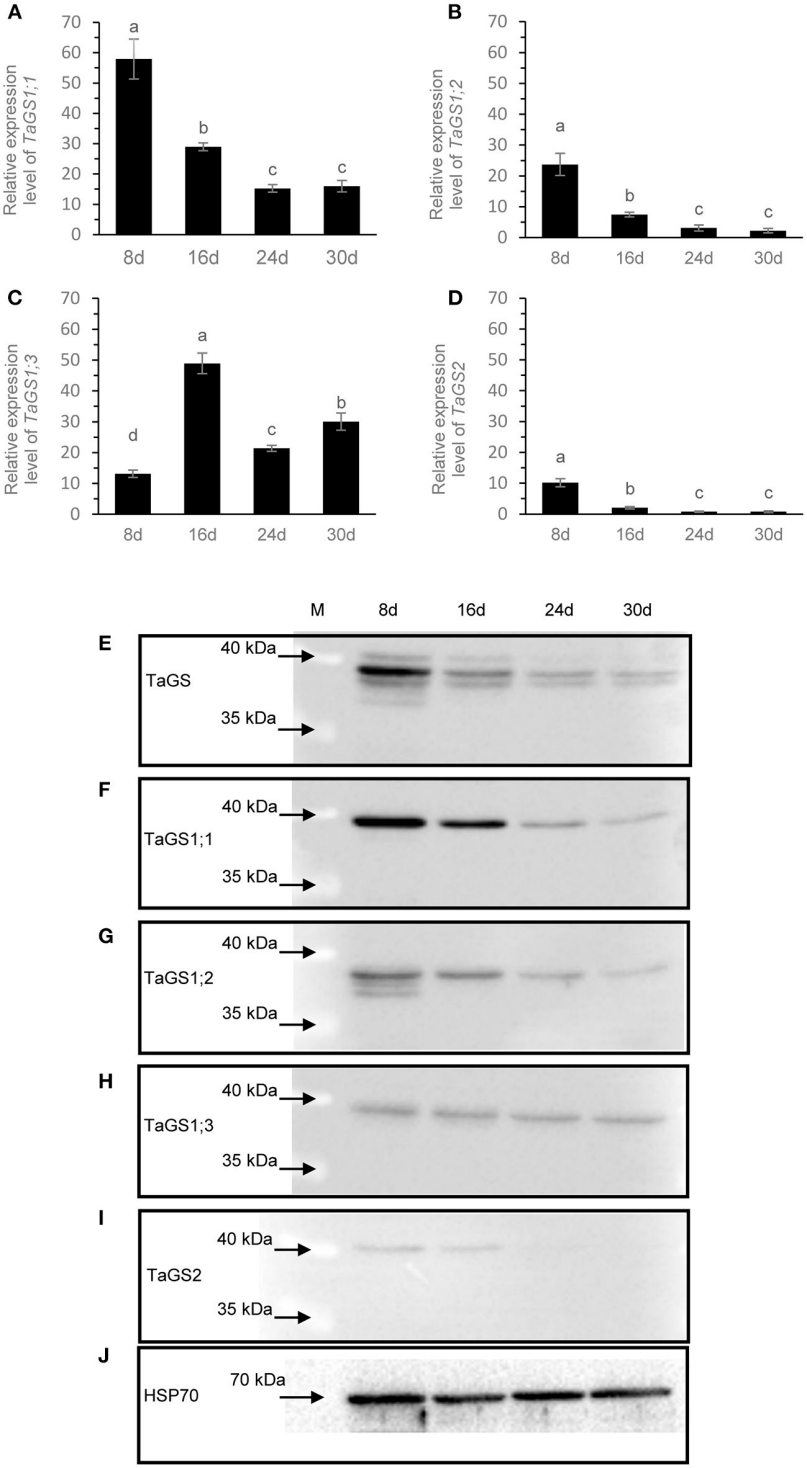
The expression profiles of TaGS isozymes in the grain during grain filling stage. Real-time PCR analysis of *TaGS1;1* **(A)**, *TaGS1;2* **(B)**, *TaGS1;3* **(C)**, and *TaGS2* **(D)** gene expression in grains during grain filling. Relative expression of each isoform normalized to reference genes *TaATPase* and *TaTEF*. Data are means of six independent biological replicates ± SD. Letters above samples indicate statistically significant differences where *p* < 0.05 according to one-way ANOVA and Duncan *post-hoc* test. Western blot analysis of expression profile of TaGSs **(E)**, TaGS1;1 **(F)**, TaGS1;2 **(G)**, TaGS1;3 **(H)**, and TaGS2 **(I)** subunits in grains during grain filling. HSP70 is used as a loading control **(J)**. A total of 20 μg of soluble proteins extracted from the seeds were loaded onto each lane and detected by the TaGS.

To further understand the roles of individual TaGS isozymes in grains during grain filling, real-time PCR and western blot were used to analyze their expression pattern. The level of TaGS1;1 mRNA was the highest among all TaGS genes at 8 DAF, while TaGS1;3 relative transcript level was the highest after 8 DAF (Figures 4A,C). TaGS1;1, TaGS1;2, and TaGS2 had similar expression patterns, with the highest expression level at 8 DAF, after which it gradually decreased (Figures 4A,B,D). However, TaGS1;3 had a different expression pattern to other TaGS genes in grain, with the lowest expression level at 8 DAF and the highest expression level at 16 DAF, after which it significantly decreased (Figure 4C).

According to the amino acid sequences of TaGS1;1 (AAZ30057.1), TaGS1;2 (AAR84347.1), and TaGS1;3 (AAR84349.1), TaGS2 (AAZ30060.1) was obtained in NCBI. The theoretical molecular mass of the TaGS1;1, TaGS1;2, TaGS1;3, and TaGS2 (no signal peptides) subunit were calculated as 39.2, 38.7, 39.5, and 41.8 kDa, respectively. The anti-TaGS antibody was used to analyze TaGS expression in wheat grains; four TaGS bands with molecular mass of about 42, 39.5, 38.5, and 36.5 kDa were detected in the grains at 8 DAF (Figure 4E). When probed with individual TaGS isoform-monospecific antibodies, the results confirmed that the subunit size of TaGS2 was 42 kDa and that of TaGS1;1 and TaGS1;3 was 39.5 kDa, but that of TaGS1;2 was 38.5, 37.5, and 36.5 kDa (Figures 4F–I). Maybe due to the higher sensitivity of TaGS1;2-monospecific antibodies compared to TaGS antibodies, a weak TaGS1;2 band with a size of about 37.5 kDa was detected (Figure 4G). The original images of western blot are shown in Supplementary Figure 2.

The expression of individual TaGS was different in the grain during grain filling phase. At 8 DAF, TaGS1;1 was the main isoform of TaGS in grain (Figures 4E,F). TaGS1;1 and TaGS1;2 had similar expression patterns, with the highest expression level at 8 DAF, after which it gradually decreased (Figures 4E–G). The expression level of TaGS1;3 isozyme was relatively stable during the grain filling stage (Figure 4H). TaGS2 was detected in grains at 8 DAF, but the amount decreased significantly at 16 DAF, and it was difficult to detect from 24 DAF (Figure 4I). These results strongly indicate that TaGS isozymes play different roles in grains during the grain filling stage.

### N Metabolism in the Grain During Grain Filling

**Figure 5:**
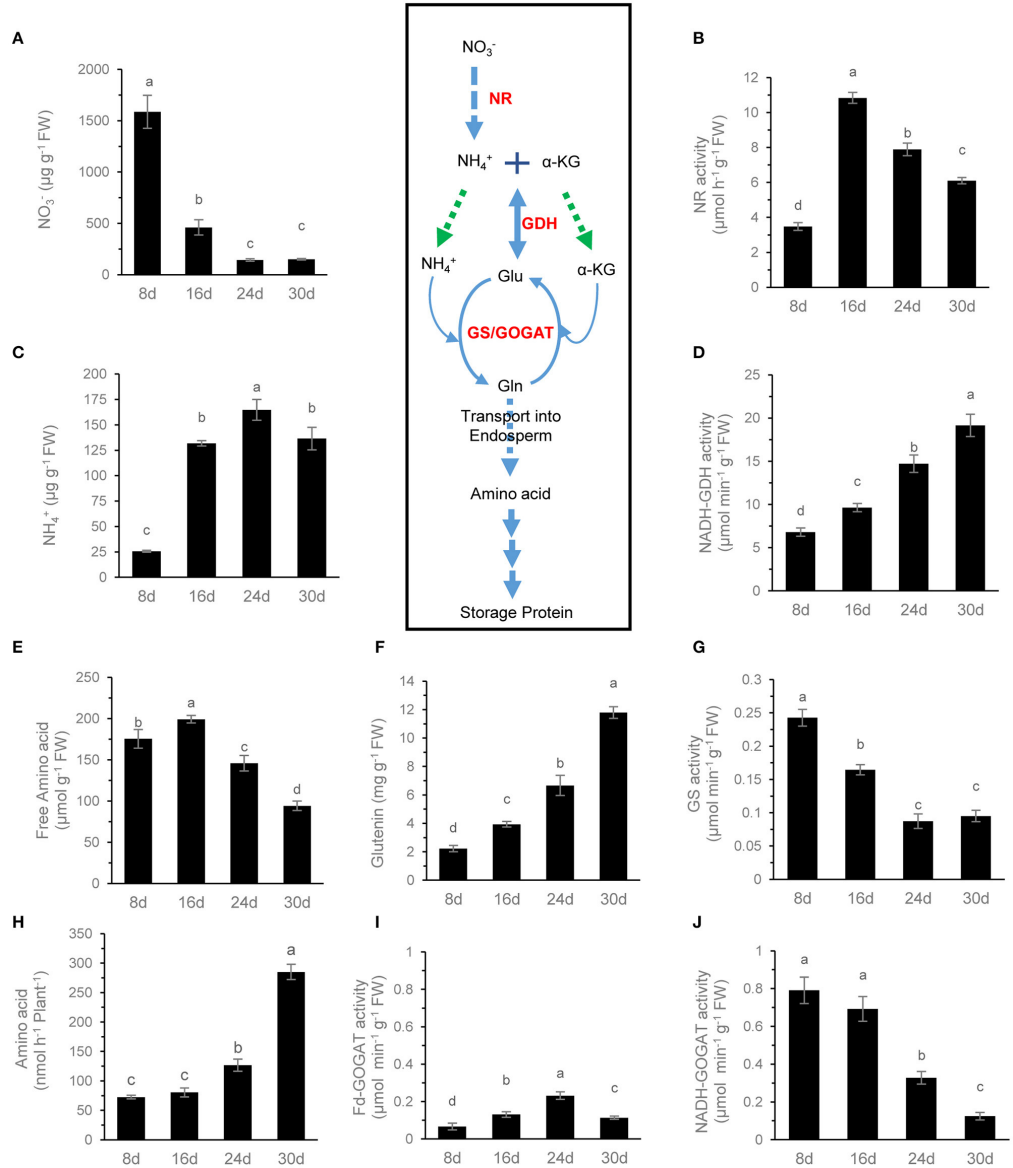
N metabolite analysis of grains during grain filling. The ${\text{NO}}_{3}^{-}$ content **(A)**, NR activity **(B)**, free ${\text{NH}}_{4}^{+}$ content **(C)**, NADH-GDH activity **(D)**, free amino acid content **(E)**, glutenin content (**F**), GS activity **(G)**, Fd-GOGAT activity (**I**), and NADH-GOGAT activity **(J)** of grains were determined. The rate of amino acid exudation from the peduncle phloem **(H)** was analyzed. Data are means of six independent biological replicates ± SD. The different letters above each sample indicate statistically significant differences where *P* < 0.05 according to one-way ANOVA and Duncan *post-hoc* test.

In wheat, ${\text{NO}}_{3}^{-}$ is the main form of inorganic N absorbed from soil and stored in the shoot, which can be reduced into ${\text{NH}}_{4}^{+}$ by nitrate reductase (NR). During grain filling, NR activity was lowest when the ${\text{NO}}_{3}^{-}$ content was highest, and the lowest ${\text{NH}}_{4}^{+}$ content in the grain occurred at 8 DAF, after which NR activity increased rapidly, resulting in a significant ${\text{NO}}_{3}^{-}$ content decrease and ${\text{NH}}_{4}^{+}$ content increase (Figures 5A–C). Therefore, ${\text{NH}}_{4}^{+}$, from the reduction of ${\text{NO}}_{3}^{-}$, became the major nitrogen resource in grains. At 16 DAF, ${\text{NH}}_{4}^{+}$ content in the grain increased rapidly up to 140 μg/g FW, about five times than that in the flag leaf of wheat (Supplementary Figure 4). The results may suggest that the ${\text{NH}}_{4}^{+}$ assimilation pathway in grains is different from that in the leaf.

In most cases, ${\text{NH}}_{4}^{+}$ content in the leaf is very low, and the GS-glutamate synthase (GOGAT) cycle plays a major role in ${\text{NH}}_{4}^{+}$ assimilation. Although Fd-GOGAT and NADH-GOGAT were present in grains (Hayakawa et al., 1994), the activity of NADH-GOGAT in wheat grains was much higher than that of Fd-GOGAT, and it was the primary GOGAT activity in grains (Figures 5I,J). During grain filling, the aminating activity of GDH increased continuously while the activity of GS and NADH-GOGAT decreased continuously and synchronously (Figures 5D,G,J). These results suggested that the GS-GOGAT cycle plays the main role of ${\text{NH}}_{4}^{+}$ assimilation in the earlier grain filling stage, while the GDH and GS-GOGAT pathways may involve in ${\text{NH}}_{4}^{+}$ assimilation in the later stage of grain filling.

**Figure 6:**
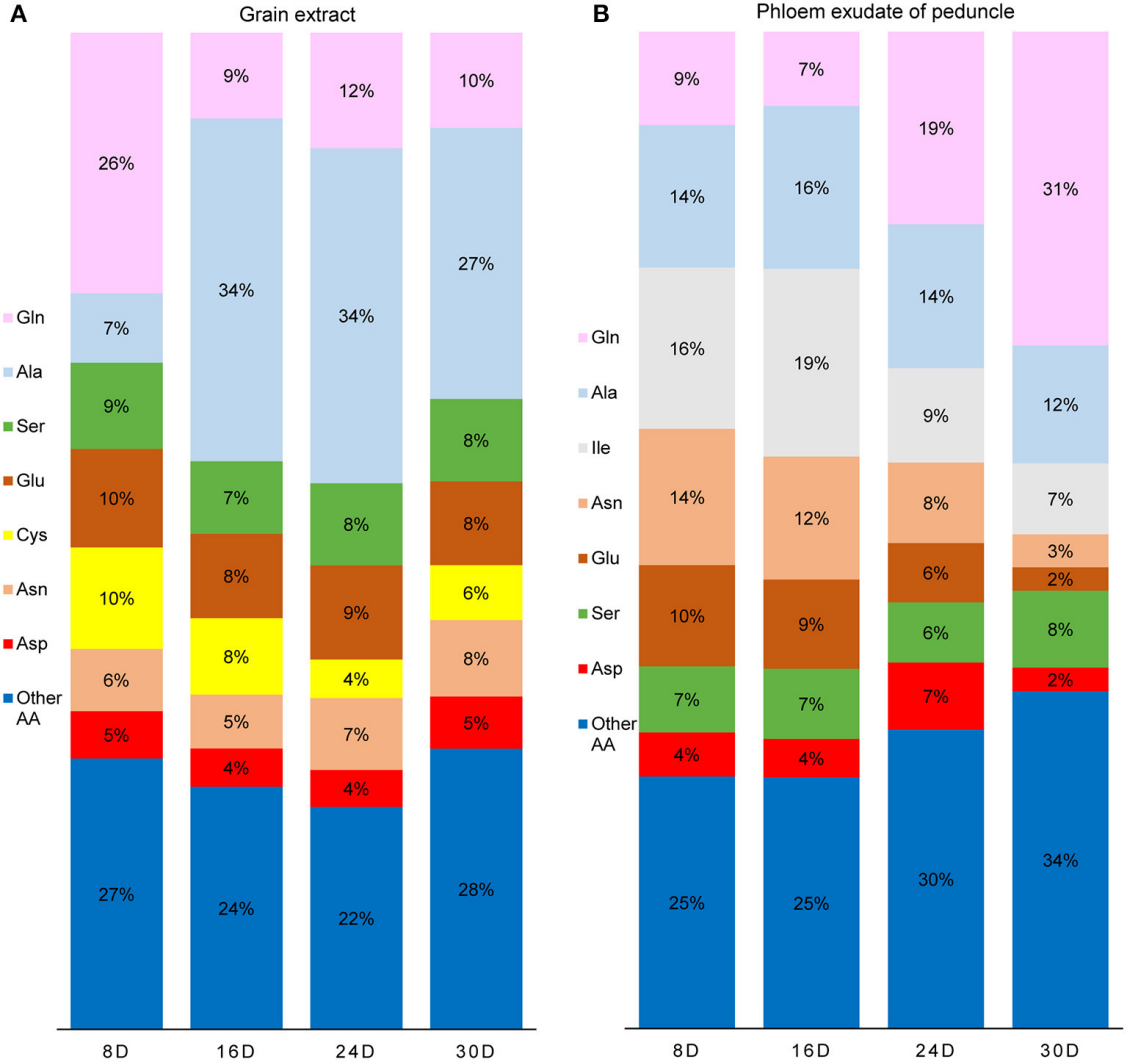
The proportion of amino acid (molar-%) in the grain extracts **(A)** and the phloem exudate of peduncle **(B)** during grain filling. Extractable amino acids of grain were 175, 199, 145, and 94 μmol g^−1^ FW, and the rate of amino acid exudation from the peduncle phloem was 72, 80, 126, and 285 nmol h^−1^ plant^−1^ at 8, 16, 24, and 30 days after flowering, respectively.

Amino acids are the main raw materials for the synthesis of storage protein in grains. The amino acids in the grain are not only from ${\text{NH}}_{4}^{+}$ assimilation but also from the input of the maternal phloem. The content of free amino acids in the phloem sap of peduncle was very low at 8 and 16 DAF but increased significantly after 24 DAF. It reached the highest level at 30 DAF, and Gln and Ala were the main amino acids at 30 DAF (Figures 5H, 6B). In contrast, the content of free amino acids in the grains remained at a high level at 8 and 16 DAF but then decreased rapidly, and Gln was the main amino acid at 8 DAF, while Ala was the main amino acid after 16 DAF (Figures 5E, 6A). Glutenin, as the main form of storage protein in grains, continuously accumulated during grain filling, and its accumulation was obviously accelerated after 24 DAF (Figure 5F). These results indicate that the amino acids used for glutenin synthesis are mainly transported from maternal phloem in the late grain filling stage.

## Discussion

GS is closely related to grain N concentration, grain size, and yield components of crops (Obara et al., 2001; Gallais and Hirel, 2004; Tabuchi et al., 2005; Martin et al., 2006; Brauer et al., 2011; Hu et al., 2018; Gao et al., 2019). Although the localization and function of the individual GS isozymes in leaves, stems, and roots have been well-studied, their distribution and role in grains remain largely unknown. In wheat grain, we discovered that TaGS isozymes were mainly located in embryo and grain transport tissue, and they synergistically performed ${\text{NH}}_{4}^{+}$ assimilation in grain.

### Cellular Localizations and Expression Profiles Suggest Distinctive Roles of TaGS Isozymes in Grain

Only *HvGS1;3* transcripts were found in the barley embryo (Goodall et al., 2013), but TaGS1;1, TaGS1;3, and TaGS2 isozymes were observed in wheat embryo (Figures 2, 3). In the embryo, proteins and nucleic acids are largely synthesized, which requires Gln as the direct or indirect organic N donor for amino acids and nucleotides (Miflin and Habash, 2002; Fu et al., 2019). In plants, Gln is mainly synthesized by GS; therefore, TaGS1;1, TaGS1;3, and TaGS2 isozymes located in embryos may participate in amino acid and nucleotide synthesis by providing Gln.

**Figure 7:**
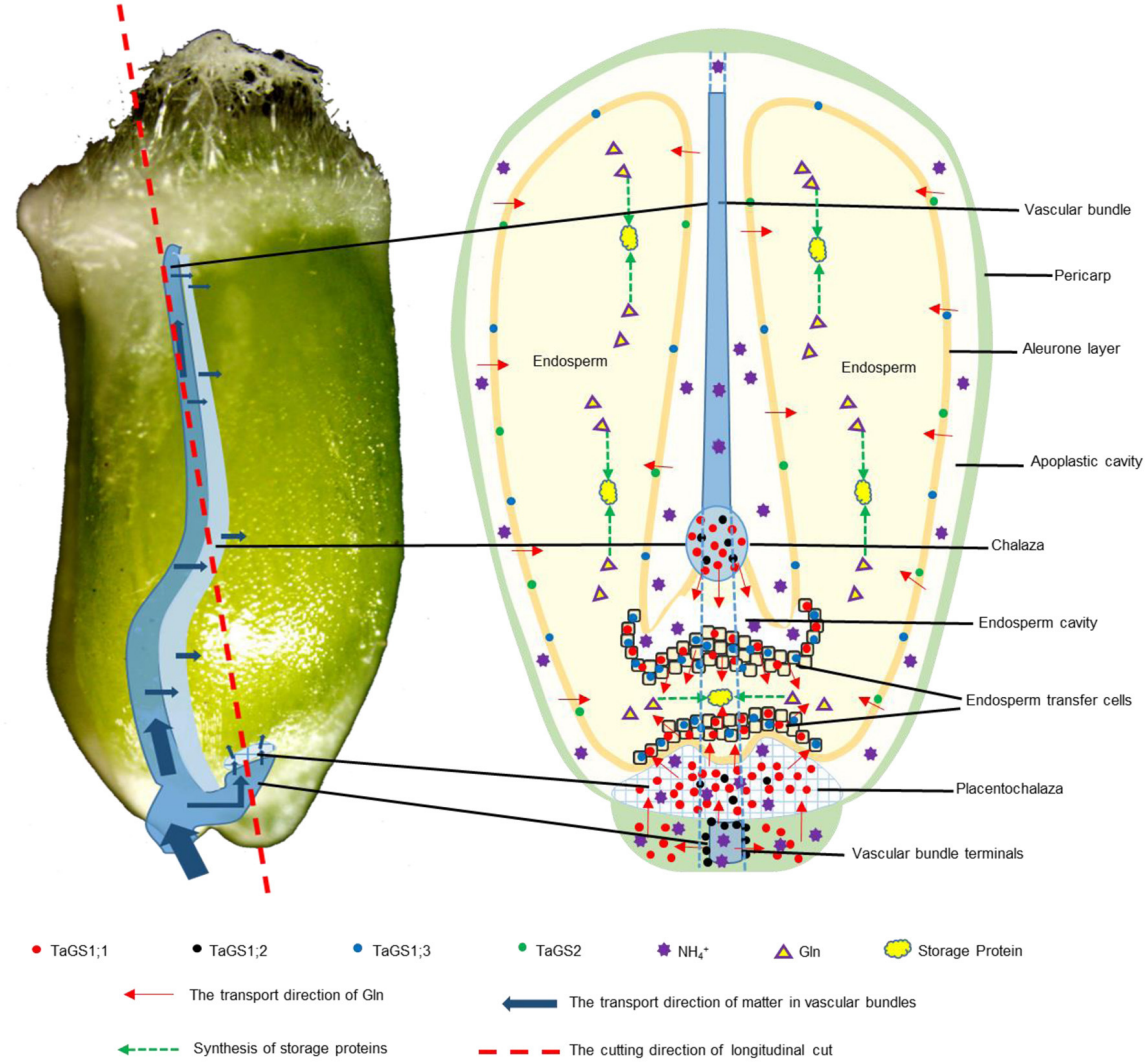
Graphical representation of the localization and function of GS in wheat grain. The direction of Gln transfer is indicated by red arrows. The transport direction of matter in vascular bundles is indicated by blue arrows. The green dotted arrow indicates the synthesis of storage proteins. The red dotted line indicates the cutting direction of longitudinal cut. The red, black, blue, and green dots indicate the localization of TaGS1;1, TaGS1;2, TaGS1;3, and TaGS2, respectively.

The vascular bundle of grain is important for assimilation to enter into filial tissues (Peukert et al., 2014). In Arabidopsis, AtGS1 isozymes localized in phloem companion cells of the vascular bundle in leaves act together for N-remobilization and grain filling (Moison et al., 2018). In wheat grain, TaGS1;2 was distributed in the phloem companion cells of vascular bundle and around the vascular bundle (Figures 2H,I); therefore, Gln biosynthesis for N-remobilization that occurred in the vascular bundle of grain may be carried out by TaGS1;2 (Figure 7).

The vascular bundle terminals below the placentochalaza are the unloading region of the assimilates (Thompson et al., 2001). In this study, vascular bundle terminals and placentochalaza were also observed in the wheat grain at 16 DAF (Figures 2, 3, 7). In previous studies, the transverse section structure in the middle of the wheat grain and the longitudinal section structure of embryos were mainly observed (Patrick and Offler, 2001; Thompson et al., 2001; Zheng, 2012; Peukert et al., 2014; Radchuk and Borisjuk, 2014). However, the placentochalaza region of wheat was located at the bottom of the seed abdomen, so it is rarely observed. TaGS1;1 and TaGS1;2 were distributed in the vascular bundle terminals (Figure 2L), suggesting that they may be involved in the assimilation of the ${\text{NH}}_{4}^{+}$ unloaded from the vascular bundle terminals (Figure 7). In tobacco, overexpression of TaGS1;1 significantly increased the length of seeds to improve N assimilation and unloading (Supplementary Figure 5).

Both chalaza and placentochalaza transport assimilates from the maternal vascular bundle into filial tissues (Zheng, 2012). GS activity was detected in the placentochalaza of maize kernels (Purcino et al., 2000). In wheat grain, TaGS1;1 and TaGS1;2 were distributed in the chalaza and placentochalaza (Figures 2B,C,K,L), indicating that they participate in the assimilation of ${\text{NH}}_{4}^{+}$ from the vascular bundle and translocation of Gln into filial tissues (Figure 7).

The main function of endosperm transfer cells is to uptake metabolite, such as sugars and amino acids, for grain filling (Thompson et al., 2001). TaGS1;1 and TaGS1;3 were distributed in the endosperm transfer cells (Figures 2B,C,3B,C), implying that Gln biosynthesis for N-remobilization occurring in ETC of grain was catalyzed by TaGS1;1 and TaGS1;3 (Figure 7).

The cross cells of the pericarp are adjacent to the apoplastic cavity, and TaGS1;1, TaGS1;3, and TaGS2 were found in cross cells (Figure 2E,3E,H), indicating that they were involved in the assimilation of ${\text{NH}}_{4}^{+}$ from the apoplastic cavity. During grain filling, aleurone layer cells transport assimilates into endosperm (Zheng, 2012). TaGS1;3 and TaGS2 were distributed in aleurone layer cells (Figures 3E,H), suggesting that glutamine biosynthesis for N-remobilization occurs in aleurone layer cells, which were catalyzed by TaGS1;3 and TaGS2 (Figure 7).

In the knockout mutants of *ZmGln1;4* of maize, kernel size is significantly reduced due to the inefficient transportation of amino acid to developing grains (Martin et al., 2006). TaGS1;1, homologous to ZmGln1;4, was distributed in the transport tissue of wheat grain, so it may play the same role in amino acid transportation during grain development.

Leaves contribute about 50% of grain N, and a large amount of amino acids are exported through phloem in the late stages of senescence of the flag leaves (Simpson and Dalling, 1981). At 30 DAF, numerous amino acids were derived from the maternal phloem and transported into filial tissue that were used to synthesize storage protein (Figures 5E,F,H), and TaGS1;3 was the highest expressed of TaGS isozyme in grains (Figure 2C). Moreover, among the four TaGS isozymes, TaGS1;3 has the highest *V*_max_ and the strongest ${\text{NH}}_{4}^{+}$ assimilation ability (Wei et al., 2020). In wheat, the QTL for grain protein content was significantly associated with GSe (TaGS1;3) (Gadaleta et al., 2014). The TaGS1;3 isozyme, located in the ETC and aleurone layer, expressed stably, indicating that TaGS1;3 plays an important role in the transportation of organic N into endosperm during the late grain filling stage (Figure 7).

At the late stage of grain filling, Gln and Ala were the main amino acids entering the grain through phloem (Figure 6B), while the content of free amino acids in the grain decreased continuously (Figures 5E,F) for large amount of storage protein synthesis. Although Ala was the main free amino acid in the grains at 30 DAF, the Ala content decreased rapidly since then (Peeters and Van Laere, 1994; Zhang et al., 1998), for the input of Ala reduced during maturation or the grain Ala can be converted by aminotransferase into Glu or Gly for protein synthesis (Zhong et al., 2018). Thus, Ala is not the main free amino acid form in mature grains (Muttucumaru et al., 2006; Curtis et al., 2009).

TaGS isozymes in the grain play different roles in the process of assimilates entering the grain, reflected by the differences in their distribution and expression.

### The Special ${\text{NH}}_{4}^{+}$ Assimilation Pathway in Wheat Grain

High concentrations of ${\text{NH}}_{4}^{+}$ in plant cells leads to ${\text{NH}}_{4}^{+}$ toxicity (Li et al., 2010; Esteban et al., 2016). During the wheat grain filling stage, the content of ${\text{NH}}_{4}^{+}$ in flag leaves was stable at around 27 μg/g FW (Supplementary Figure 4), which was similar to the content of ${\text{NH}}_{4}^{+}$ (25 μg/g FW) in grains at 8 DAF (Figure 5C). The content of ${\text{NH}}_{4}^{+}$ in grains reached 140 μg/g FW after 16 DAF (Figure 5C). It would be useful to determine how to avoid ${\text{NH}}_{4}^{+}$ toxicity in the embryo and endosperm cells. Sun et al. (2016) found that both ${\text{NH}}_{4}^{+}$ and ${\text{NO}}_{3}^{-}$ in rice grains are mainly distributed in the outer layer of the grains, and the amount gradually decreased moving inwards. Therefore, most ${\text{NH}}_{4}^{+}$ in grains may be distributed in the pericarp and the apoplastic cavity to avoid the toxicity of high concentration of ${\text{NH}}_{4}^{+}$ to the embryo and endosperm tissues. At 16 DAF, apoplastic cavity was observed between the pericarp and endosperm (Figures 2A,D) and may contain a lot of ${\text{NH}}_{4}^{+}$.

Another issue to address is how to assimilate the high concentration of ${\text{NH}}_{4}^{+}$ in the grain. In plant, ${\text{NH}}_{4}^{+}$ is mainly assimilated into organic nitrogen by GS-GOGAT cycle. After 16 DAF, there was a high ${\text{NH}}_{4}^{+}$ concentration in the grains (Figure 5C), but the activity of GS and NADH-GOGAT decreased with grain filling (Figures 5G,J), while the aminating activity of GDH increased (Figure 5D). These results indicated that the grains may have a special ${\text{NH}}_{4}^{+}$ assimilation pathway.

GDH incorporates ${\text{NH}}_{4}^{+}$ into 2-oxoglutarate to form glutamate or oxidize glutamate. Although the ^15^N- or ^13^C-labeling experiments have clearly demonstrated that the deamination reaction occurs in the cell, it has been argued that under certain physiological conditions, when the ${\text{NH}}_{4}^{+}$ concentration reaches a certain threshold, the GDH is involved in ${\text{NH}}_{4}^{+}$ assimilation (Dubois et al., 2003). So, it will be interesting to study the role of GS, GOGAT, and GDH in grains with high concentration of ${\text{NH}}_{4}^{+}$.

## Data Availability Statement

The original contributions presented in the study are included in the article/Supplementary Material, further inquiries can be directed to the corresponding author/s.

## Author Contributions

YW, SX, XW, and XMa planned and designed the research. YW, ZZ, XMe, LW, XZ, and MY performed experiments, conducted fieldwork, and analyzed data. YW, HY, and XW wrote the manuscript. YW and SX contributed equally. All authors read and approved the final version of this manuscript.

## Conflict of Interest

The authors declare that the research was conducted in the absence of any commercial or financial relationships that could be construed as a potential conflict of interest.
