# Supplementary material for: Localization, Gene Expression, and Functions of Glutamine Synthetase Isozymes in Wheat Grain (Triticum aestivum L.)
Source: Front Plant Sci. 2021 Feb 9;12:580405. doi: 10.3389/fpls.2021.580405 (PMC7901976; doi:10.3389/fpls.2021.580405)
Supplement: Supplementary file 1 [file Table_1.DOCX]

Table S1. Meteorological data of growth seasons on air temperature and rainfall.

| Month | Average air temperature | Rainfall |
| --- | --- | --- |
|  | (℃) | (mm) |
| Oct-2017 | 14.64 | 34.9 |
| Nov-2017 | 8.64 | 5.8 |
| Dec-2017 | 1.8 | 1 |
| Jan-2018 | -1.35 | 7.1 |
| Feb-2018 | 2.94 | 2.3 |
| Mar-2018 | 6.05 | 14.7 |
| Apr-2018 | 16.48 | 167 |
| May-2018 | 22.13 | 84.1 |
| June-2018 | 21.94 | 80.4 |

Table S2 Primers used to amplify the cDNA of *TaGS1;1*, *TaGS1;2*, *TaGS1;3* and *TaGS2* from wheat by RT-PCR

| Gene Name | Primer | Sequence(5'-3') |
| --- | --- | --- |
| *TaGS1;1* | *TaGS1;1-F* | ACCCGCCTTCCTTCCTGC |
|  | *TaGS1;1-R* | CGATGATGCGACCTACCTAAGC |
| *TaGS1;2* | *TaGS1;2-F* | CATTCCCTCCTTGCGAG |
|  | *TaGS1;2-R* | AAATGGAAACACGAAACG |
| *TaGS1;3* | *TaGS1;3-F* | GAAGAAGAAGAAGAGGTAGCCATG |
|  | *TaGS1;3-R* | AACAGAACCCATCAAAGCCAC |
| *TaGS2* | *TaGS2-F* | GCGGAGTAAGTAAGTAAGCAGC |
|  | *TaGS2-R* | CATGCGGAGCGGTTCTAC |

Table S3 Primers used to amplify the CDS of *TaGS1;1*, *TaGS1;2*, *TaGS1;3* and *TaGS2* from wheat

| Gene Name | Primer | Sequence(5'-3') |
| --- | --- | --- |
| *TaGS1;1* | *TaGS1;1-F* | CTTTAAGAAGGAGATATACATATGGCGCTCCTCACCGATCTCC |
|  | *TaGS1;1-R* | TGCTCGAGTGCGGCCGCAAGCTTGGGCTTCCACAGGATGGTGGTCTC |
| *TaGS1;2* | *TaGS1;2-F* | CTTTAAGAAGGAGATATACATATGGCCAGCCTCGCCGACC |
|  | *TaGS1;2-R* | TGCTCGAGTGCGGCCGCAAGCTTGAGGAGGAGCGTGGTCTCGGCG |
| *TaGS1;3* | *TaGS1;3-F* | CTTTAAGAAGGAGATATACATATG TCTCCGCTCGCCGACCTTC |
|  | *TaGS1;3-R* | TGCTCGAGTGCGGCCGCAAGCTTCTTGCCATTGGAGAGACCGGCC |
| *TaGS2* | *TaGS2-F* | CTTTAAGAAGGAGATATACATATGCTCGGCCCGGAGACCACCG |
|  | *TaGS2-R* | TGCTCGAGTGCGGCCGCAAGCTTTACCTTCAGCGCCAGCTTCTTGGC |

Table S4 List of primers used for qPCR.

| Gene Name | Primer | Sequence(5'-3') |
| --- | --- | --- |
| *TaGS1;1* | *TaGS1;1-F* | AAGGACGGCGGGTTC AA |
|  | *TaGS1;1-R* | GCGATGTGCTCCTTGTGCTT |
| *TaGS1;2* | *TaGS1;2-F* | GACAACTTCCTTGTTATGTGCCAC |
|  | *TaGS1;2-R* | TGTGCCTCTTGTTCGTGGG |
| *TaGS1;3* | *TaGS1;3-F* | CTG TGA CTG CTA TGC GCC TAA C |
|  | *TaGS1;3-R* | CCG CGT TGT ACC GCT TGT |
| *TaGS2* | *TaGS2-F* | GGT TGA CAG GGC TAC ACG AGA |
|  | *TaGS2-R* | GAG CAG CCA CGG TTC GC |
| *ATPase* | *ATPase-S* | ATACGCCATCAGGGAGAACATC |
|  | *ATPase-A* | AGGGTTGTCCTTCCTCCGC |
| *TaTEF1* | *TaTEF1-S* | GGTTGTGGAGACCTTTGCTACTTAC |
|  | *TaTEF1-A* | AACAGCCACAGTTTGCCTCAT |
