# Supplementary material for: Localization, Gene Expression, and Functions of Glutamine Synthetase Isozymes in Wheat Grain (Triticum aestivum L.)
Source: Front Plant Sci. 2021 Feb 9;12:580405. doi: 10.3389/fpls.2021.580405 (PMC7901976; doi:10.3389/fpls.2021.580405)
Supplement: Supplementary file 2 [file Image_1.PDF]

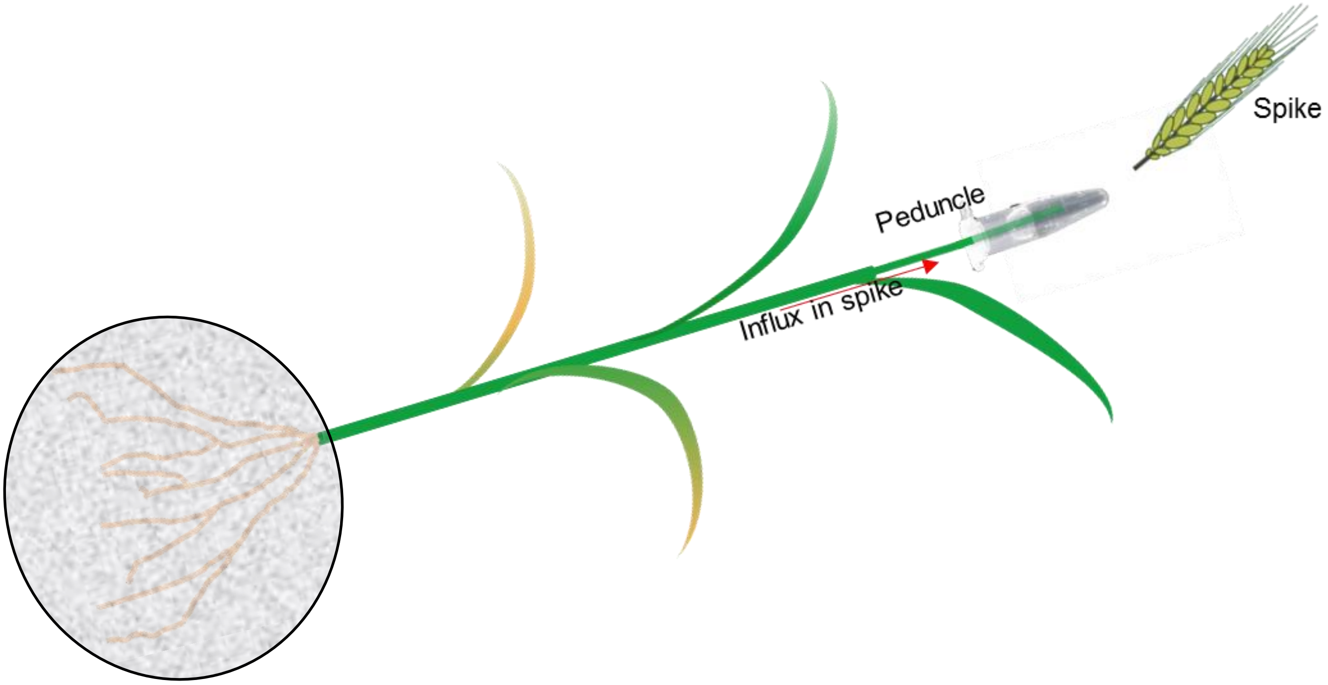

Figure S1 The method to obtain the phloem exudate of peduncle. The wheat plants are exposed to high humidity and darkness during the collection process.

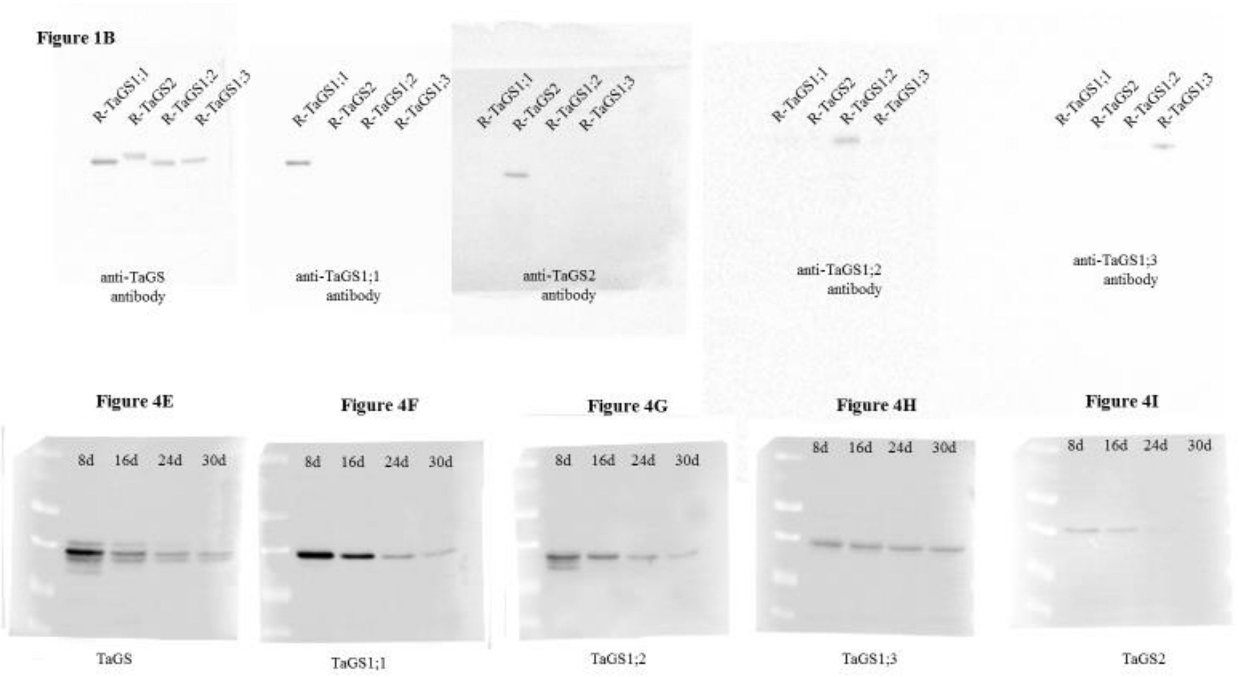

Figure S2 The western blot figures of Figure 1B and Figure 4E-I which has not been trimmed.

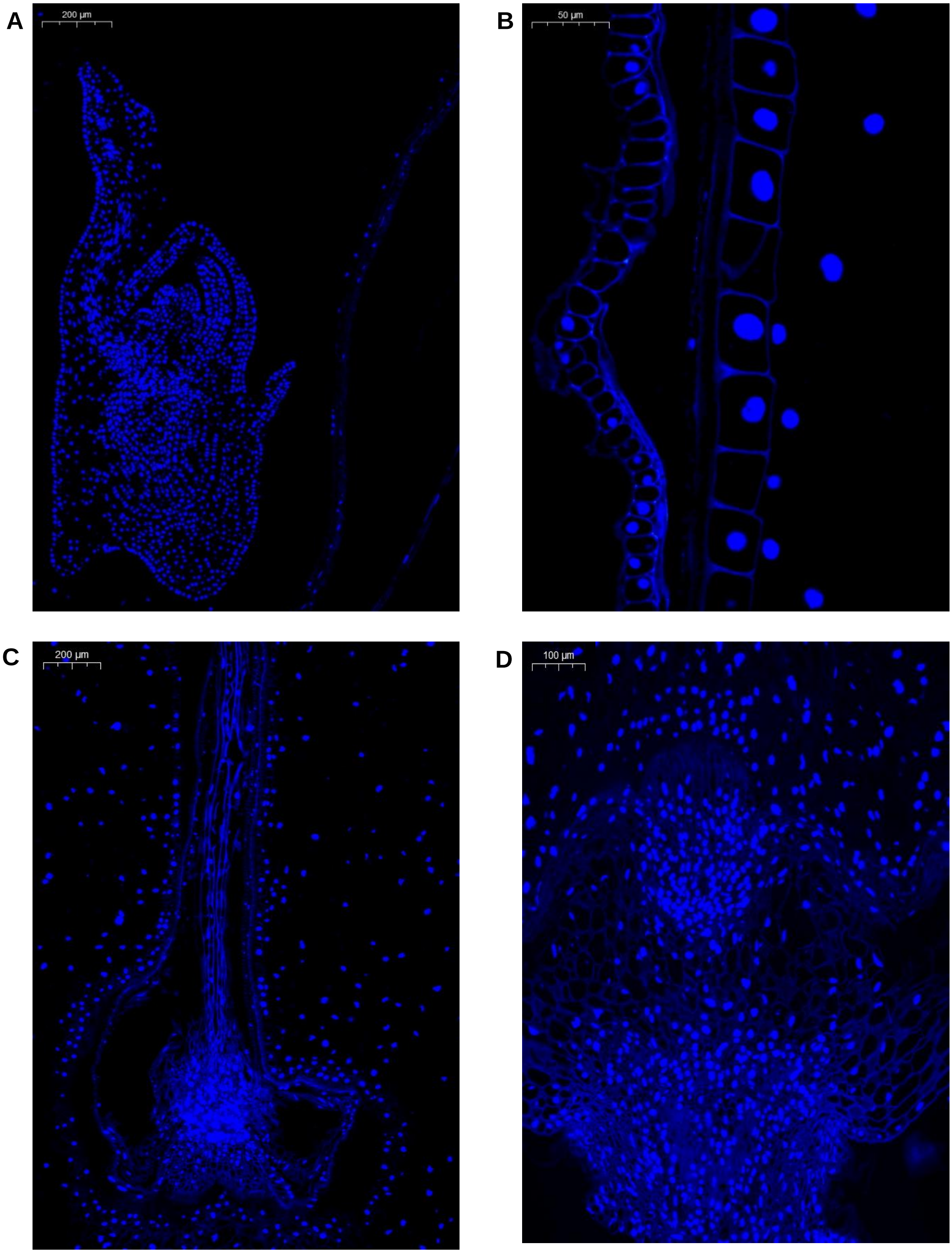

Figure S3 Negative controls section of embryo (A), aleurone layer (B), chalazal region (C), and placentochalaza region (D) treated with preimmune rabbit serum.

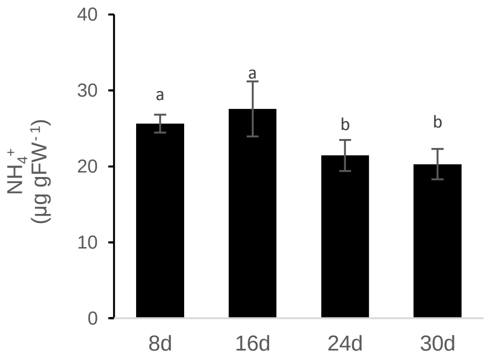

Figure S4 The content of NH_4^+ in the flag leaves during the grains filling. Data are means of six independent biological replicates ± SD. The different letters above each sample indicate statistically significant differences where $P < 0.05$ according to one-way ANOVA Duncan post-hoc test.

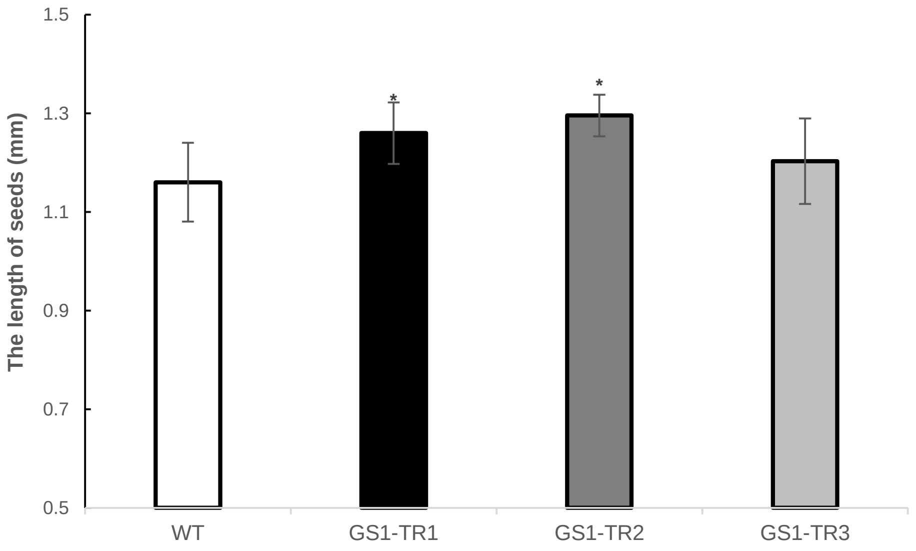

Figure S5 The length of seeds harvested from the wild-type (WT) and overexpression TaGS1;1 tobacco. Three independent transformed lines, GS1-TR1, GS1-TR2, and GS1-TR3, were used to analyzed. Values are means ±SE (n=30) of seed length. Asterisks indicate that the data is significantly different ($p < 0.05$) from the data of WT plants.
